# Gold nanorod-based smart platform for efficient cellular uptake and combination therapy†

**DOI:** 10.1039/d4ra06051b

**Published:** 2024-08-28

**Authors:** Kibeom Kim, Mamta Ramgopal Chejara, Been Yoon, Myoung-Hwan Park

**Affiliations:** a Department of Chemistry and Life Science, Sahmyook University Seoul 01795 South Korea mpark@syu.ac.kr; b Department of Convergence Science, Sahmyook University Seoul 01795 South Korea; c Convergence Research Center, Nanobiomaterials Institute, Sahmyook University Seoul 01795 South Korea

## Abstract

Gold nanorods (GNRs) have received much attention as potential drug-delivery vehicles because of their various advantages such as good biocompatibility, passive targeting, responsiveness to stimuli, and easy post-functionalization by surface modification. However, the drug structure might be changed for loading into GNRs, making it difficult to load various drugs, and the space to contain drugs is small, making it difficult to deliver sufficient drugs required for treatment compared with other porous materials. Herein, we report an amphiphilic polymer-coated GNR platform for chemo- and photothermal combination therapy. Amphiphilic polymers comprise hydrophobic alkyl chains for drug encapsulation, polyethylene glycol for biocompatibility, and folic acid for cancer targeting. GNRs generate heat energy under near-infrared light irradiation, promoting controlled drug release, and inducing cellular uptake by deforming the cell membrane. On-demand release behavior was traced with Nile red, and targeting and delivery efficiency were confirmed with paclitaxel through cellular experiments. This GNR-based platform enables combination therapy with passive and active targeting to enhance the efficacy of cancer treatment.

## Abstract 2

An amphiphilic polymer-coated GNR platform was studied for chemo- and photothermal combination therapy, promoting controlled drug release, and inducing cellular uptake by deforming the cell membrane under NIR light.

## Graphical abstract

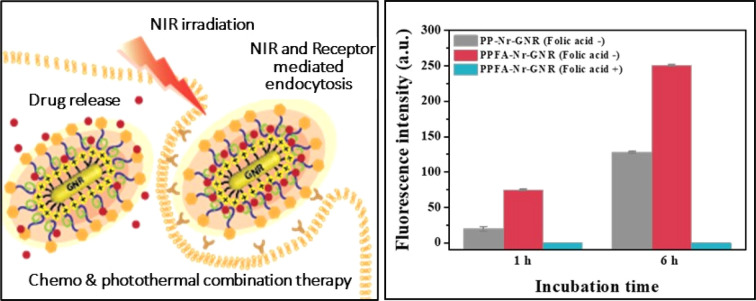

## Introduction

Cancer is defined as irregular abnormal cells that aggressively invade and metastasize; it is the cause of death of millions of people.^1^ Although chemotherapy is the most widely used cancer treatment method, the direct administration of anticancer drugs has challenges, such as drug solubility and side effects.^2–4^ Therefore, to overcome these challenges, drug delivery systems based on silica nanoparticles, quantum dots, polymer nanoparticles, metal–organic frameworks, and metal nanoparticles have been developed over the past few decades.^5–13^ Among them, gold-based drug delivery systems are widely researched as drug delivery system structures because of various advantages such as biocompatibility, responsivity to stimuli, and easy functionalization through surface modification.^8,14^ In particular, the gold nanorod (GNR), a smart material, generates heat *via* the surface plasmon resonance (SPR) effect by near-infrared (NIR) light, which has the advantage of being able to control drug release or provide a variety of treatment methods.^15–17^ However, in contrast with porous materials, which load the drug into their pores, GNRs are linked with the drug by covalent bonds, which introduces specific functional groups that modify the GNRs.^18–20^ Therefore, it is difficult to use various drugs for cancer treatment. In addition, the chemical structure of a drug is modified by the introduction of a linker, which can cause a change in the unique properties of the drug.^21–23^ To overcome this challenge, a polymer or mesoporous shell, which provides the space for loading the various drugs to nanoparticles, was introduced on the surface of GNRs.^24–27^ These modification methods allow for drug loading, increasing therapeutic effects, but the encapsulated drug amount is still limited compared to systems based on other mesoporous materials.^28–31^ Therefore, to increase the cancer therapy effect based on GNRs as a drug delivery vehicle, a novel platform that can load various drug types without structural modification and induce drug delivery into cancer cells to compensate for the low loading amount is needed.

In this paper, a novel platform composed of GNRs and an amphiphilic polymer shell is presented. A ligand with a trimethylammonium functional group was modified using a previously reported method, and positively charged GNRs were synthesized. A polymer (PP) composed of palmitic acid and polyethylene glycol was synthesized and a folic acid functional group which facilitate the cancer targeting was added to synthesize an amphiphilic polymer (PPFA) (Scheme S1†). PP and PPFA are modified on the surface of GNRs by electrostatic interactions, and various drugs can be loaded into the hydrophobic part of the amphiphilic polymer without changing the chemical structure of the drug. The ethylene glycol group not only imparts a negative charge to the polymer but also imparts high biocompatibility and reticuloendothelial system (RES)-evading properties to the platform.^32,33^

**Scheme 1:**
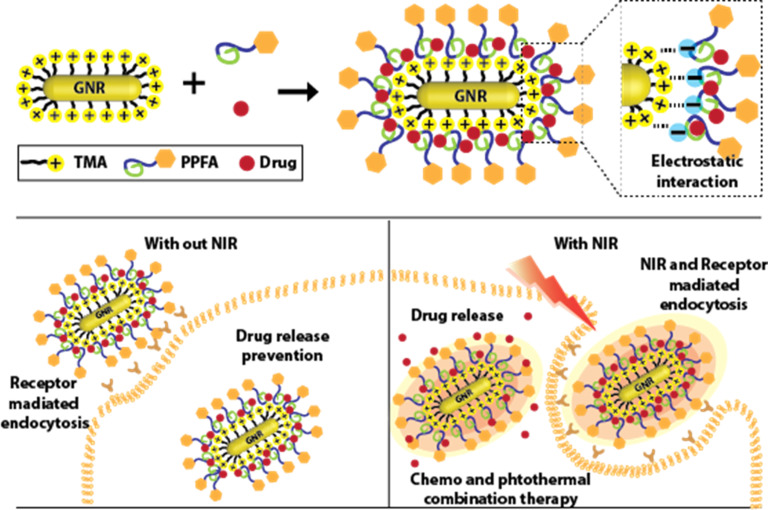
Illustration of preparation of GNRs-based platform and therapy procedure.

In addition, folic acid facilitates cancer-targeted drug delivery by selectively interacting with folate receptors overexpressed in cancer cells.^34–38^ The heat energy, which is generated by the SPR effect of GNRs under NIR light irradiation, facilitates the controlled release of the loaded drug on the platform to maintain the concentration of the drug within the therapeutic window, thereby reducing side effects.^15,39–41^ In addition, the cancer therapeutic effect can be increased through combination therapy.^42–46^ Furthermore, the generated heat energy induces the cellular uptake rate of the platform and increases the intracellular concentration of the drug.^47^ This approach is one step closer to precision medicine, providing multifunctional capabilities, including cancer-targeting, increased cellular uptake of drugs, controlled drug release, and combination therapy (Scheme 1).

## Experimental section

### Materials and methods

#### General information

Chloroauric acid tetrahydrate (HAuCl_4_·4H_2_O ≥ 99.9%), hexadecyltrimethylammonium bromide (CTAB ≥ 96%), sodium borohydride (NaBH_4_, 99%), silver nitrate (AgNO_3_ ≥ 99.9%), l-ascorbic acid (AA ≥ 99.0%), 11-bromo-1-undecanol (98%), triphenyl methanethiol (97%), methane sulfonyl chloride (MSc, 98%), trifluoroacetic acid (≥99%), triisopropylsilane (98%), trimethylamine (≥99.5%), triethylamine (≥99%), folic acid-dialysis membrane (10 kDa), 4-dimethlaminopyridine (DMAP, ≥99%), dimethyl sulfoxide, *N*-(3-dimethylaminopropyl)-*N*′-ethylcarbodiimide hydrochloride (EDC), *N*,*N*′-dicyclohexylcarbodiimide (DCC, 99%), folic acid (≥97%), and formamide were purchased from Sigma-Aldrich. Nile red (Nr) was purchased from TCI and used as a fluorescent probe for the release test and cellular uptake studies. Deionized (DI) water was produced by the Millipore Milli-Q system (18.2 mΩ cm). Paclitaxel crystalline solid (PTX, ≥99.5%) and polyethylene glycol bis amine (PEG, 2000 g mol^−1^) were purchased from Alfa Aesar. Palmitic acid (PA) was purchased from Acros Organics. All cell reagents used for *in vitro* studies, phosphate-buffered saline (PBS), Dulbecco's modified Eagle's medium (DMEM), fetal bovine serum (FBS), and penicillin–streptomycin were purchased from Sigma-Aldrich. Cell viability was quantified using a resazurin cell proliferation assay (ready-to-use solution) purchased from TCI.

#### Preparation of CTAB-GNRs

CTAB-GNRs were synthesized using a seed-mediated growth method, employing initially, a seed solution was prepared by combining 7.5 mL of 0.1 M CTAB, 250 μL of 0.01 M HAuCl_4_, and 800 μL of 0.01 M NaBH_4_, followed by stirring for 2 to 3 h to obtain a brown-yellow solution. A growth solution containing 1.8 mL of 0.01 M HAuCl_4_, 180 μL of 0.01 M AgNO_3_, and 288 μL of 0.1 M AA, along with 130 μL of the seed solution, was incubated at 35 °C for 24 h. Excess CTAB was removed from the GNRs by centrifugation at 13 000 rpm.

#### Preparation of TMA-GNRs

Thiolated cationic trimethyl ammonium (TMA) ligands were synthesized by following previously reported methods.^48^ TMA ligands were attached to gold nanorods (GNRs) through Au–S bonds using a one-pot ligand exchange method, conducted in deionized water. For the TMA-GNRs preparation, 0.25 mL of a 10 mM TMA solution was gradually added to 7.5 mL of the GNRs solution, with the mixture then stirred gently for 72 h. To purify the TMA-modified GNRs, the mixture was centrifuged at 13 000 rpm for 20 min, separating the excess TMA ligands from the GNRs.

#### Synthesis of PP polymer

The PP was synthesized using EDC coupling strategy. Palmitic acid (0.5 g, 0.0019 mol) was activated by EDC (0.795 g, 0.0038 mol) and DMAP (0.476 g, 0.0038 mol) for 30 min. After activation, PEG (5.01 g, 0.00507 mol) was added by dropwise into activated palmitic acid solution and stirred for 12 h at room temperature. Final product of PP was purified by column chromatography. The chemical structures of the synthesized molecules were confirm ^1^H NMR (Fig. S3†). ^1^H NMR (400 MHz, DMSO-D6) *δ*-0.855 (t, 3H), *δ*-1.235 (s, 26H), *δ*-1.476 (t, 2H), *δ*-2.038 (t, 2H), *δ*-3.180 (t, 2H), *δ*-3.420 (m, 45H), *δ*- 7.82 (S, 1H).

#### Synthesis of PPFA polymer

The PPFA was synthesized using DCC coupling strategy. Free carboxyl group in terminal of folic acid was attached to amine group of PEG using DCC/DMAP coupling mechanism. DCC (22 mg, 0.11 mmol) and DMAP (13 mg, 0.1 mmol) was added to DMSO solution which consist of folic acid (40 mg, 0.11 mmol) and stirred for 1 h to activate the carboxyl group of FA. PP (50 mg, 0.025 mmol) was added to the activated FA and stirred for 24 h at room temperature. Final product PPFA was purified by column chromatography. The chemical structures of the synthesized molecules were confirm ^1^H NMR (Fig. S5†). ^1^H NMR (400 MHz, DMSO-D6) *δ*-0.870 (t, 3H), *δ*-1.234 (s, 26H), *δ*-1.477 (t, 2H) *δ*-2.038 (m, 4H) *δ*-2.289 (t, 2H) *δ*-3.476(m, 45H) *δ*-3.510 (t, 2H) *δ*-4.294 (t, 2H) *δ*-4.479 (s, 2H) *δ*-4.605 (m, 1H) *δ*-6.631 (d, 2H), *δ*-67.640 (d, 2H) *δ*-8.652 (s, 1H).

#### Preparation of Nr-loaded PPFA-GNRs (PPFA-Nr-GNR)

A solution of TMA-GNRs with a concentration of 20.3 nM and a volume of 3.5 mL was slowly added to a mixture solution containing PPFA (10 mg, 3.7 mmol) and Nr (3 mg, 9.47 mmol), under dark conditions. This mixture was stirred continuously for 24 hours. PPFA was modified on the TMA-GNRs by electrostatic interaction. Following stirring, the solution underwent dialysis using a 14 kDa membrane against a solution of 1% DMSO to remove Nr and PPFA. After the dialysis process, the product was freeze-dried to obtain the final compound.

#### Preparation of PTX-loaded PPFA-GNRs (PPFA-PTX-GNRs)

A solution of TMA-GNRs with a concentration of 20.3 nM and a volume of 3.5 mL was slowly added to a mixture solution containing PPFA (10 mg, 3.7 mmol) and PTX (3 mg, 0.035 mmol), under dark conditions. This mixture was stirred continuously for 24 hours. PPFA was modified on the TMA-GNRs by electrostatic interaction. Following stirring, the solution underwent dialysis using a 14 kDa membrane against a solution of 1% DMSO to remove PTX and PPFA. After the dialysis process, the product was freeze-dried to obtain the final compound.

#### Evaluation of thermal elevation efficiency of PPFA-Nr-GNRs

PPFA-Nr-GNRs at various concentrations (0.25, 0.5, 1, and 2.5 nM) were exposed to NIR light at a power of 1.6 W cm^−2^ for a duration of 15 minutes, with temperature variations recorded using an infrared camera. Additionally, to evaluate the impact of varying NIR light intensities, a PPFA-Nr-GNR solution at 2.5 nM concentration was subjected to different laser powers (0.6, 1.1, and 1.6 W cm^−2^), with the resulting temperature changes also monitored through an infrared camera.

#### Release experiment of PPFA-Nr-GNRs

To study the release of Nr triggered by NIR light, PPFA-Nr-GNRs were exposed to NIR light at intensities of 0.6, 1.1, and 1.6 W cm^−2^ in a PBS. The released Nr was quantified using fluorescence spectroscopy.

#### Competitive inhibition of cellular uptake after saturation of FR receptor

The specific affinity of PPFA-Nr-GNRs towards the folate receptor was confirmed by pre-treating HeLa cells with free folic acid to block these receptors. After a 2 h incubation, the cells were washed twice with serum-free medium. PP-Nr-GNRs and PPFA-Nr-GNRs were introduced to the cells, which were then incubated for 1 and 6 h. Post-incubation, the cells wash before the Nr fluorescence was observed using confocal laser scanning microscopy (CLSM). The fluorescence intensity was analyzed with ImageJ software.

#### NIR light-mediated cellular uptake of PP-Nr-GNRs and PPFA-Nr-GNRs

To investigate NIR light-induced cellular uptake, HeLa cells were plated at a density of 1 × 10^4^ cells per well in 12-well plates with DMEM supplemented with 10% FBS and incubated for 24 h 37 °C in a 5% CO_2_ environment. PP-Nr-GNRs and PPFA-Nr-GNRs were introduced to the cells, which were then exposed to NIR light at 1.6 W cm^−2^ for 1, 5, and 10 min. Post-irradiation, the cells were washed three times with serum-free DMEM. The uptake of Nr was observed using CLSM, and quantified using ImageJ software.

#### Cell viability studies using MTT assay

To assess the therapeutic efficacy and biocompatibility of the nanoplatforms, cell viability of HeLa cells was determined using the MTT assay. HeLa cells were plated at a density of 3 × 10^3^ cells per well and incubated for 24 h. The nanoplatforms PP-GNRs, PPFA-GNRs, PP-PTX-GNRs, and PPFA-PTX-GNRs were then introduced to the cells at various concentrations (0.31, 0.62, 1.25, 2.5, and 5 nM) and incubated for an additional 24 h. Cell viability was measured using the MTT assay post-incubation. For evaluating the synergistic therapeutic effect, the cells were also exposed to NIR light at 1.6 W cm^−2^ for 1, 5, and 10 min during the incubation period.

#### Live and dead assay using propidium iodide (PI) and fluorescein diacetate (FDA)

HeLa cells were cultured at a density of 5 × 10^3^ cells per well in a 12-well plate, followed by the addition of prepared PP-PTX-GNRs and PPFA-PTX-GNRs. A treated group was exposed to NIR light at a power of 1.6 W cm^−2^ for 10 min. Post-NIR irradiation, cell viability was assessed using PI/FDA double staining (PI at 2.5 μM and FDA at 5 μM). The stained cells were then imaged using CLSM, and fluorescence intensity was analyzed with ImageJ software.

## Results and discussion

GNRs are widely used as drug delivery systems because they generate heat in response to near-infrared stimuli, and their surfaces can be easily modified with various ligands and thiol groups. GNRs, which were used at this platform, were synthesized by seed-mediated method. Then, CTAB, which formed a bilayer arrangement on the surface of the GNRs to stabilize the nanoparticles, was exchanged with the TMA ligand by a robust Au–S bond to endow the GNRs with high colloidal stability. In addition, TMA-ligand-modified GNRs (TMA-GNRs) exhibit a positive charge density, facilitating electrostatic interactions to immobilize negatively charged PPFAs. After the PPFA modification, Nile red was loaded onto the GNRs as a model drug (PPFA-Nr-GNR). This functionalization was characterized using ultraviolet-visible (UV-vis) spectroscopy, zeta potential analysis, and transmission electron microscopy (TEM). After ligand exchange with the TMA ligand, the UV-vis spectrum of the GNRs showed redshifts with a broadened longitudinal surface plasmon resonance (LSPR) at 760 nm compared to that of the CTAB-GNRs, which was around 745 nm. PPFA modification led to a substantial red shift in LSPR to 800 nm.

**Fig. 1:**
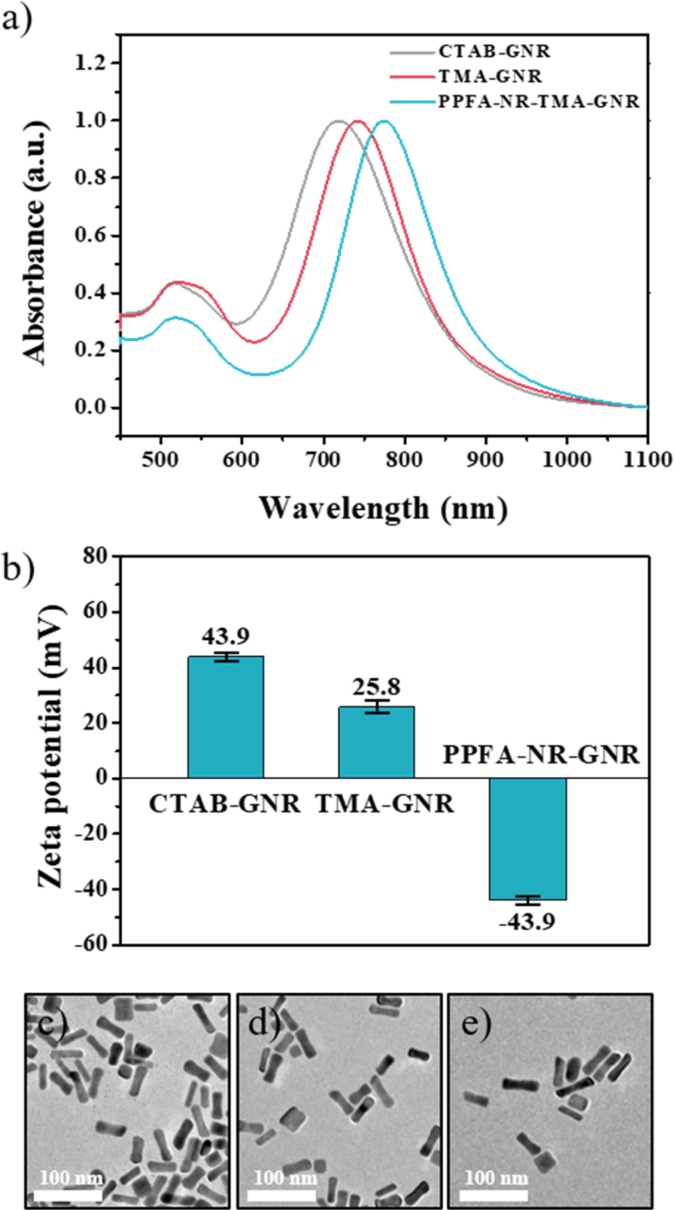
(a) UV-vis spectra graph and (b) zeta potential data. TEM images of (c) CTAB-GNRs, (d) TMA-RGNRs, and (e) PPFA-Nr-GNRs.

The exchange of ligands on the GNRs surface changes the dielectric constant of the GNR environment, causing a redshift in the LSPR. Meanwhile, an insignificant change in the UV-vis graph was observed with model drug loading. This is thought to be due to the overlap with the transverse surface plasmon resonance (TSPR) of the GNR at 500–600 nm (Fig. 1a). In addition, a change in the surface charge of the GNRs according to the functionalization process was observed based on the zeta potential. The ligand exchange to TMA leads to a charge decrease in the GNRs from 45.7 mV to 25.2 mV. The surface charge of PPFA-Nr-GNRs was reduced from 25.2 mV to −36.9 mV by the negative charge of PEG (Fig. 1b).

In addition, compared with CTAB-GNR, TMA-GNR, and drug-loaded PPFA-Nr-GNR, no significant change in the morphology of the GNRs was observed, and all GNRs showed sizes of 45 nm for the long axis and 10 nm for the short axis (Fig. 1c–e).

**Fig. 2:**
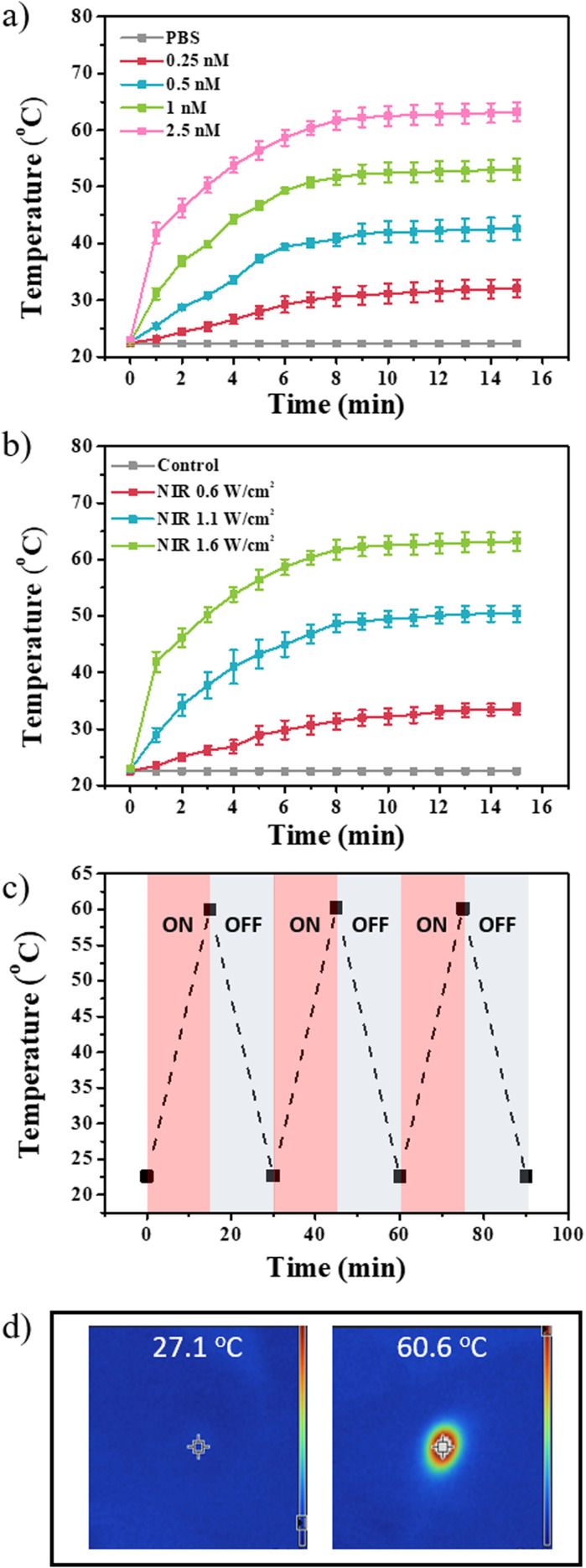
Temperature change graph of PPFA-GNR with different (a) concentration (0.25, 0.5, 1, 2.5 nM) under the light irradiation (1.6 W cm^−2^) and (b) light power (0.6, 1.1, 1.6 W cm^−2^) with predetermined concentration 2.5 nM. (c) Temperature change graph and (d) infrared camera image of the PPFA-GNR solution by the on–off of the NIR light irradiation.

UV-vis, zeta potential, and TEM data showed that ligand exchange, post-functionalization of PPFA, and drug loading were successfully achieved. To observe the response of the platform to NIR light, PPFA-GNRs were diluted in PBS, and the temperature change under NIR light was observed using an infrared camera. The temperature change was observed at 1 minute intervals from 0 to 15 min. The PPFA-GNR solutions were irradiated with NIR light at a power of 1.6 W cm^−2^. The temperatures of the solutions with concentrations of 0.25, 0.5, 1, and 2.5 nM were increased to 32.07, 42.67, 53.07, and 63.20 °C, respectively. In addition, it was confirmed that the maximum temperature reached more than 90% within 8 min of NIR light irradiation at all concentrations. Meanwhile, an insignificant temperature change was observed in the PBS solution without PPFA-GNRs (Fig. 2a).

Temperature changes can be controlled not only by concentration but also by varying the power of the NIR light. The irradiation of the 2.5 nM PPFA-GNRs solution with NIR light of 0.6, 1.1, and 1.6 W cm^−2^ power increased the solution temperature to 33.57, 50.43, and 63.20 °C, respectively (Fig. 2b). Furthermore, the temperature change of the PPFA-GNR solution by the on–off NIR light irradiation was observed, and the temperature was measured every 15 min (Fig. 2c).

The temperature of the solution approximately increased to 60 °C after NIR light irradiation and decreased to 22 °C in its absence. Fig. 2d shows each temperature change in the infrared camera image.

The data imply that heat generation is controlled by the concentration of PPFA-GNRs and the power of the irradiated NIR light. Moreover, this suggests that PPFA-GNR maintains its novel properties even after repeated NIR light irradiations.

**Fig. 3:**
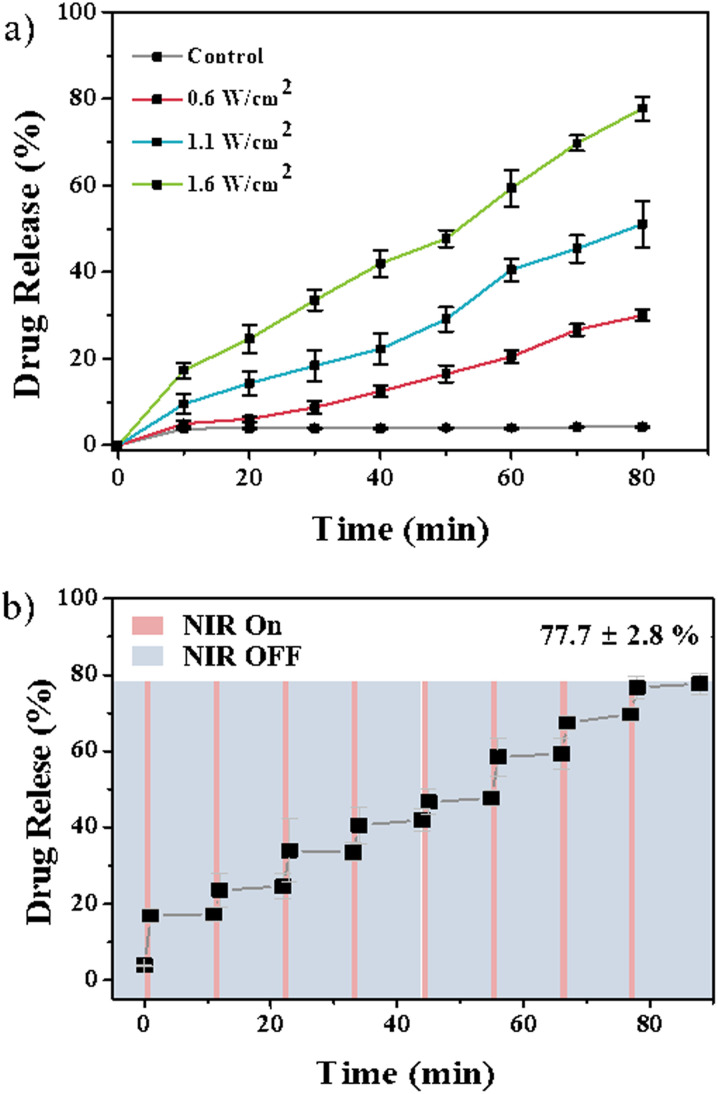
Drug release profile of PPFA-Nr-GNR with (a) different light power (0.6, 1.1, 1.6 W cm^−2^) and (b) the on–off of the NIR light irradiation.

Nr, a model drug, was initially loaded onto PPFA-GNRs to proceed with the drug release experiment. The released Nrs were measured using a fluorescence spectrophotometer to observe the drug release profiles. In the absence of NIR light irradiation, 4.25% of the loaded drug was released. However, when the drug-loaded PPFA-GNRs were irradiated with NIR light at a power of 0.6, 1.1, and 1.6 W cm^−2^, the released amount of Nr was 30.02, 51.03, and 77.73% of the loaded amount, respectively (Fig. 3a).

This implied that drug release was triggered by NIR light irradiation and that drug release is controlled by laser power. In addition, the sequential release profiles of the drugs were observed after on–off NIR light (1.6 W cm^−2^) irradiation. The interval between each sequence was approximately 11 min. NIR light was irradiated for 1 min, and the drugs were released for 10 min in the absence of NIR light irradiation. After the on–off drug release test, which lasted for 88 min, it was confirmed that approximately 77.70% of the loaded drugs were released (Fig. 3b). These results suggest that the drug concentration can be maintained within the therapeutic window by controlling drug release through on–off NIR light irradiation.

Receptor-mediated cellular uptake was observed in HeLa cells using confocal microscopy. Folic acid facilitates folate receptor-mediated endocytosis and promotes cellular uptake. Therefore, PPFA-GNRs selectively target cancer cells by interacting with overexpressed folate receptors. To confirm the increase in cellular uptake due to the interaction between folic acid and folate receptors, PPFA-Nr-GNRs and PP-Nr-GNRs without folic acid functional groups were prepared, incubated with HeLa cells, and observed for 1 and 6 h. In HeLa cells incubated with PP-Nr-GNRs for 1 h, fluorescence of Nr was not observed, and the weak fluorescence was observed after 6 h.

**Fig. 4:**
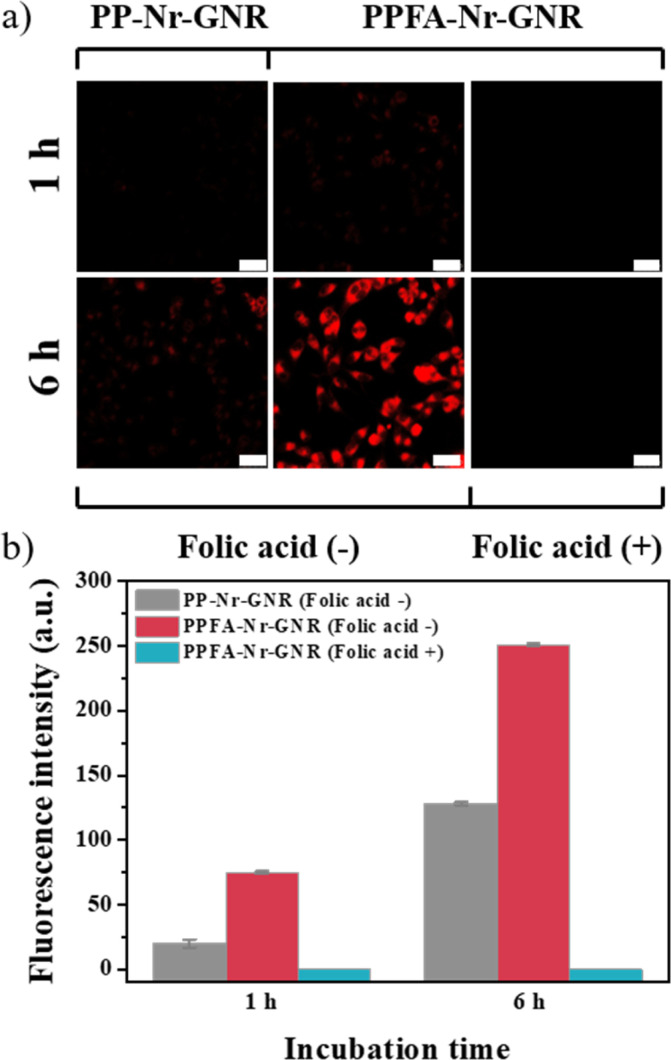
(a) Confocal images and (b) fluorescence intensity profile to check receptor-mediated cellular uptake (scale bar is 50 μm).

In contrast, weak fluorescence was observed in cells incubated with PPFA-Nr-GNRs for 1 h, and furthermore, strong fluorescence was observed after 6 h (Fig. 4a). The fluorescence intensities of the PP-Nr-GNR and PPFA-Nr-GNR treatment groups were quantified using ImageJ to compare the cellular uptake.

After 1 h incubation, the fluorescence intensity of the PPPFA-Nr-GNR group was approximately 3.7 times stronger than that of the PP-Nr-GNR group, and at 6 h, the fluorescence intensity of the PPFA-Nr-GNR group was more than 2.0 times stronger than that of the PP-Nr-GNR group (Fig. 4b). In both 1 h and 6 h incubations, the fluorescence intensity of the PPFA-Nr-GNR group was stronger, but the difference in fluorescence intensity decreased over time because of the increased fluorescence intensity of the PP-Nr-GNR group, which was internalized into cells through pinocytosis. In addition, it was confirmed that fluorescence was not observed when PPFA-Nr-GNR was incubated with HeLa cells pre-incubated with free folic acid to saturate the folate receptors, regardless of time (Fig. 4). This phenomenon is thought to occur because the folate receptors saturated by free folic acid are blocked, thus disturbing the receptor-mediated endocytosis of PPFA-Nr-GNRs. These experimental results indicate that PPFA-Nr-GNR is effectively internalized into the cell by interacting with the folate receptor overexpressed in cancer cells, which can selectively deliver drugs to cancer cells.

To confirm the change in cellular uptake by NIR light, PP-Nr-GNRs, and PPFA-Nr-GNRs were incubated with HeLa cells, irradiated with NIR light for 1, 5, and 10 min, and observed using confocal microscopy. Regardless of the NIR light irradiation time, cells in all groups were incubated for only 10 min, which was insufficient for nanoparticle endocytosis.

**Fig. 5:**
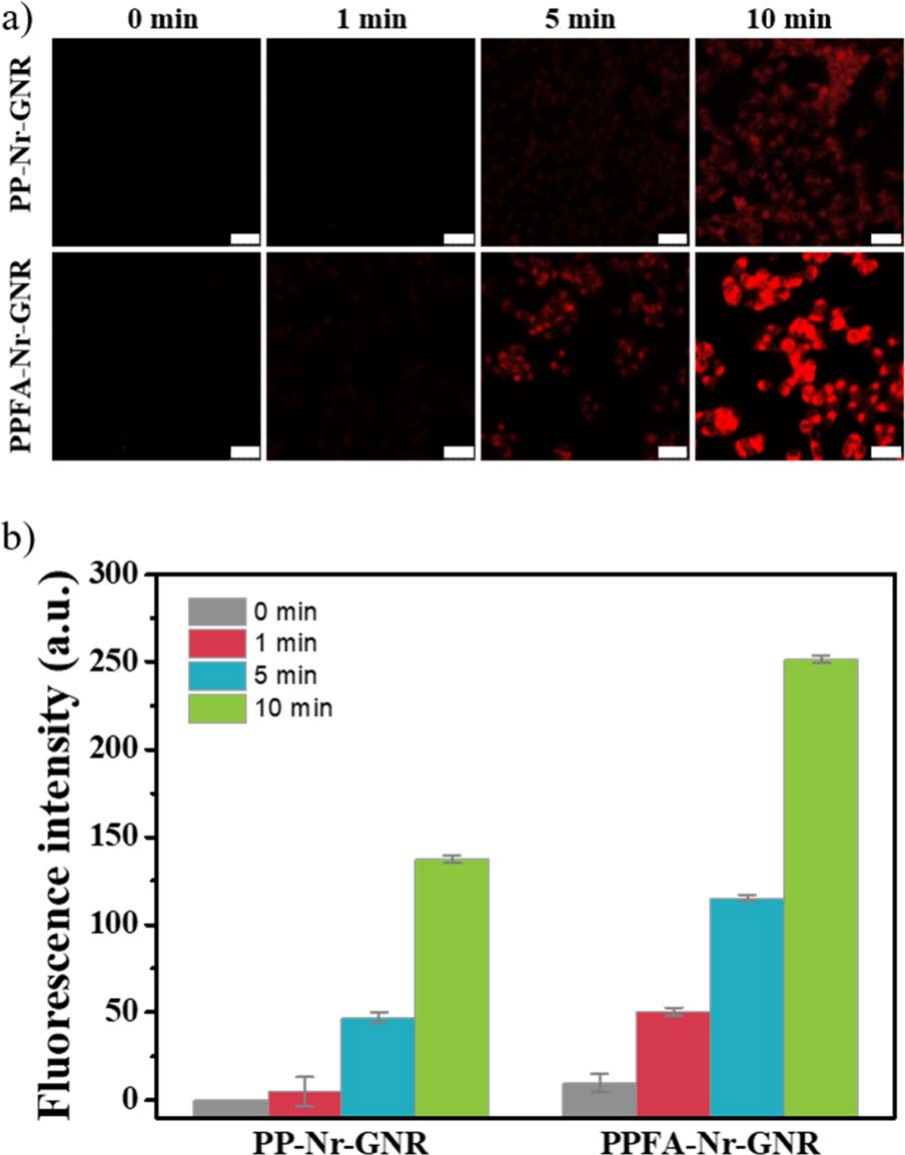
(a) Confocal images and (b) fluorescence intensity profile to check NIR light-induced cellular uptake (scale bar is 50 μm).

Therefore, the fluorescence intensity of Nr was insignificant in both groups without NIR irradiation. In contrast, in the NIR irradiation group, it was confirmed that intracellular uptake increased regardless of the presence of folic acid and that an increase in NIR irradiation time induced the intracellular uptake of GNRs (Fig. 5a).

The fluorescence intensity of the PPFA-Nr-GNR group was higher than that of the PP-Nr-GNR group. This is because folic acid induces endocytosis of GNRs by interacting with folate receptors overexpressed in cancer cells, even under NIR light irradiation. When irradiated with NIR light for 10 min, the fluorescence intensity of PP-Nr-GNR and PPFA-Nr-GNR groups increased compared to that without NIR light irradiation (Fig. 5b). The SPR effect induced by NIR light irradiation generates local heat and increases the temperature of the GNR surface, which causes strong deformation of the surrounding membrane and leads to a wrapping effect, which is an endocytic process. Therefore, cellular uptake of the platform was increased upon irradiation with NIR light.^47^

**Fig. 6:**
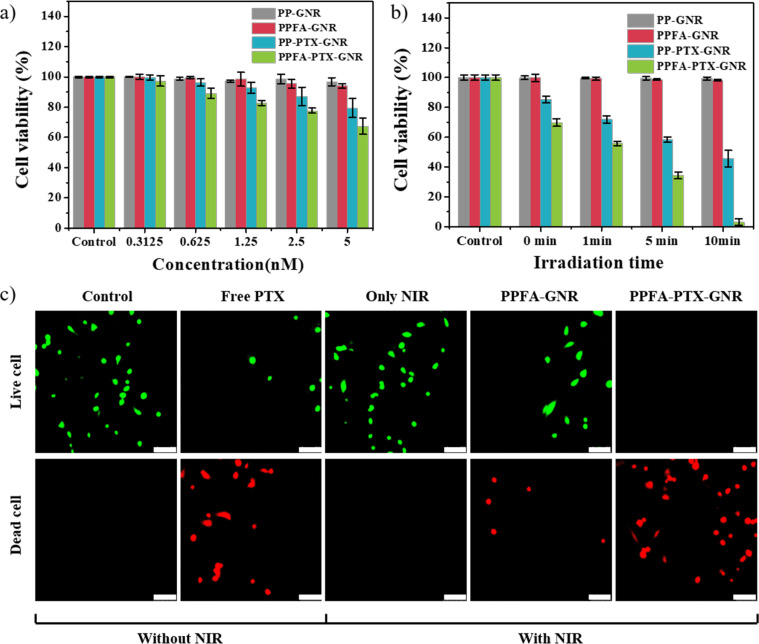
Cell viability analysis with different (a) concentrations and (b) NIR light irradiation time. (c) Confocal images of live and dead cell assays to observe the cancer therapy effect (scale bar is 75 μm).

For *in vitro* experiments, a paclitaxel (PTX)-loaded platform (PPFA-PTX-GNR) was prepared, and the biocompatibility, cancer-targeting effect, and synergistic effect of combination therapy were observed. As shown in Fig. 6a, PP-GNR and PPFA-GNR groups without drug loading show high cell viability of almost 100%. Meanwhile, the PTX-loaded platform showed decreased cell viability, and at 5 nM, the cell viability of PPFA-PTX-GNR was 67.42%, which was lower than that of PP-PTX-GNR, which had a cell viability of 79.43%. This was thought to be internalized by PTX in the cells, which was increased by receptor-mediated endocytosis. However, the amount of drug loaded onto the platform is limited and insufficient to inhibit cancer cell growth.

To overcome this challenge, NIR light was used for irradiation, and it was confirmed that cancer cell growth was suppressed as the irradiation time increased in all groups. Under NIR light irradiation for 10 min, PP-GNRs, PPFA-GNRs, PP-PTX-GNRs, and PPFA-PTX-GNRs exhibited cell viability of 99.37, 98.13, 45.67, and 3.08%, respectively (Fig. 6b). To visualize cell viability after 24 h of incubation, a live/dead cell assay was performed on HeLa cells. Live cells (green fluorescence) were observed in the control groups, NIR light-irradiated group, and PPFA-GNRs with NIR light irradiation groups and a small number of dead cells (red fluorescence) were observed in the free PTX groups. Only dead cells were observed in the group irradiated with NIR light after the PPFA-PTX-GNR treatment. The live/dead cell assay results correlated with those of the cell viability test. These experimental results indicate that the increased cellular endocytosis rate and chemo–photothermal combination therapy with PPFA-PTX-GNR increased the effect of cancer therapy.

## Conclusions

In summary, we developed a dual-targeting drug delivery system using amphiphilic polymer-coated GNRs for chemo-and photothermal combination therapies. GNRs not only have high stability but also generate heat in response to NIR radiation, which promotes photothermal therapy and endocytosis of nanoparticles.

The amphiphile polymers introduced with folic acid-loaded cancer drugs through hydrophobic interactions with the palmitic acid and polyethylene glycol moieties increase the biocompatibility of the platform. In addition, folic acid facilitates cancer targeting by interacting with folic acid receptors that are overexpressed in cancer cells. The release of the drug loaded onto PPFA-PTX-GNR was controlled by NIR light irradiation. Through *in vitro* experiments, the NIR light, receptor-mediated endocytosis, and combination therapy effects of PPFA-PTX-GNRs were confirmed. This platform effectively overcomes the inherent challenge of GNRs, which limits the amount of drug loading through dual targeting and combination therapy and expands the role of GNRs as drug carriers.

## Data availability

The data supporting this article have been included as part of the ESI.†

## Author contributions

This manuscript was written with contributions from all the authors. All the authors approved the final version of the manuscript.

## Conflicts of interest

There are no conflicts to declare.

## Supplementary Material

RA-014-D4RA06051B-s001
