# Supplementary material for: Gold nanorod-based smart platform for efficient cellular uptake and combination therapy
Source: RSC Adv. 2024 Aug 28;14(37):27385–93. doi: 10.1039/d4ra06051b (PMC11350633; doi:10.1039/d4ra06051b)
Supplement: RA-014-D4RA06051B-s001 [file RA-014-D4RA06051B-s001.pdf]

Supplementary Information (SI) for RSC Advances.
This journal is © The Royal Society of Chemistry 2024

Supplementary information

Gold nanorod-based smart platform for efficient cellular uptake and combination therapy

Kibeom Kim,[† a] *Mamta Ramgopal Chejara*,[† a, b, c] *Been Yoon*[b,c] *and Myoung-Hwan Park,*[* a, b, c]

a. Department of Chemistry and Life Science, Sahmyook University, Seoul 01795, South Korea

b. Department of Convergence Science, Sahmyook University, Seoul 01795, South Korea

c. Convergence Research Center, Nanobiomaterials Institute, Sahmyook University, Seoul 01795, South Korea

[†] These authors contributed equally to this work

*Corresponding Author: E-mail: mpark@syu.ac.kr

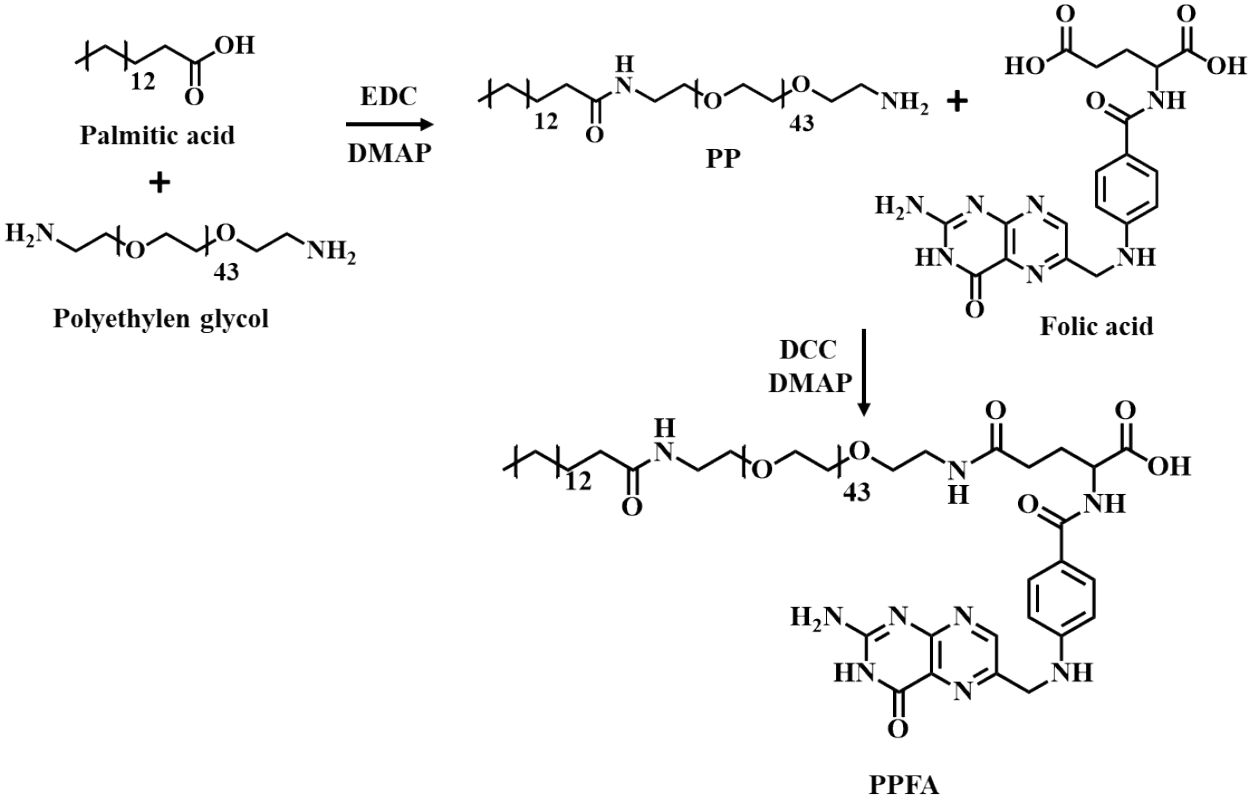

Scheme S1. Synthesis scheme of PPFA.

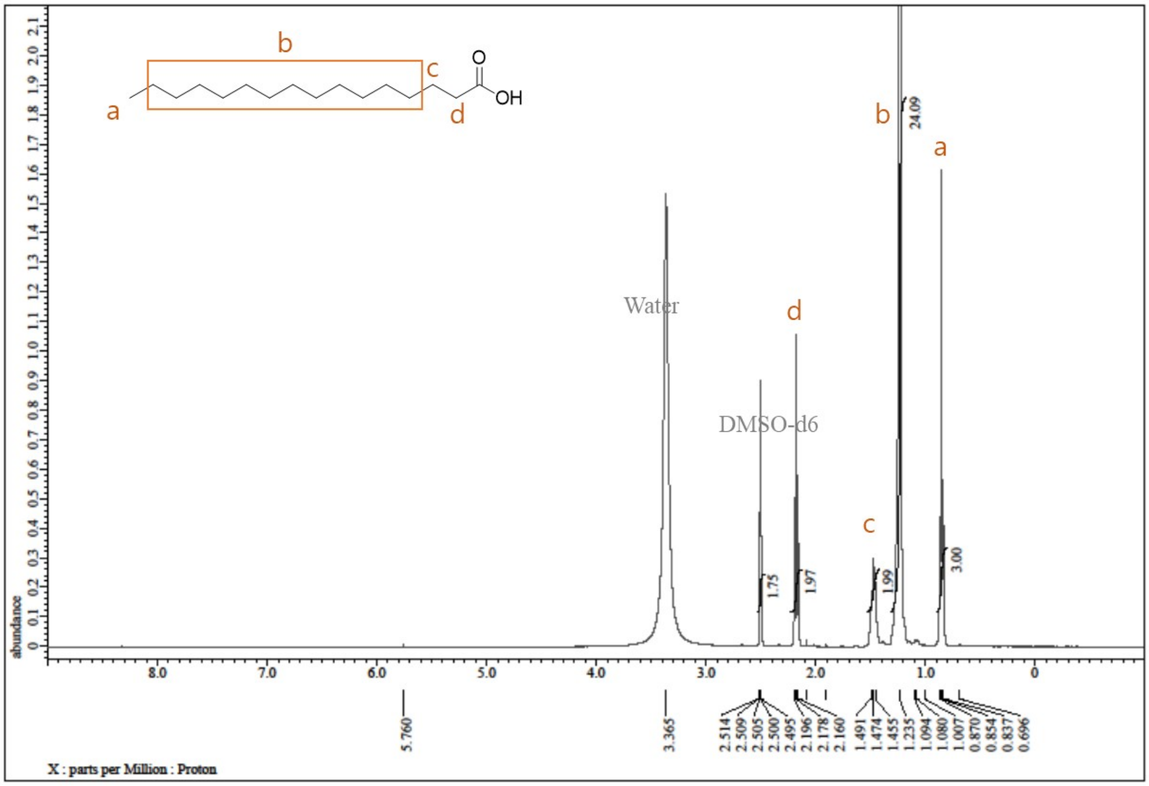

Figure S1. ^{1}H NMR spectral data of palmitic acid

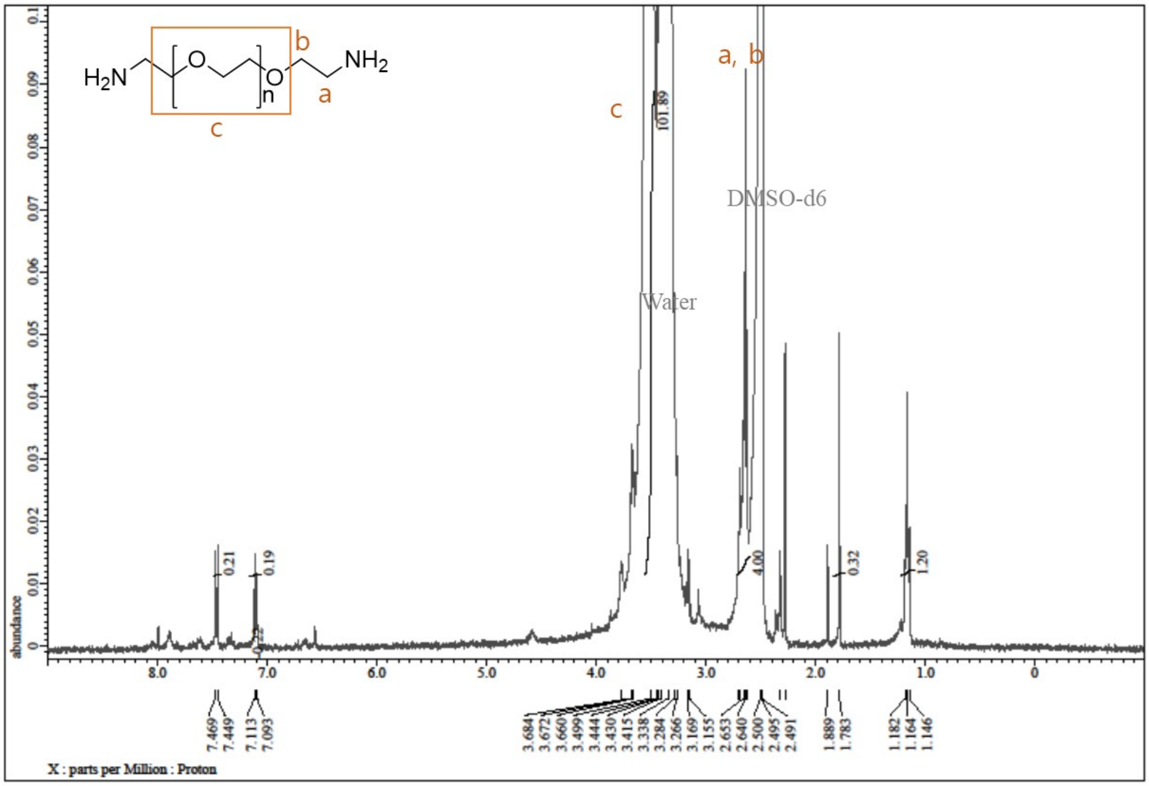

Figure S2. ^{1}H NMR spectral data of polyethylene glycol

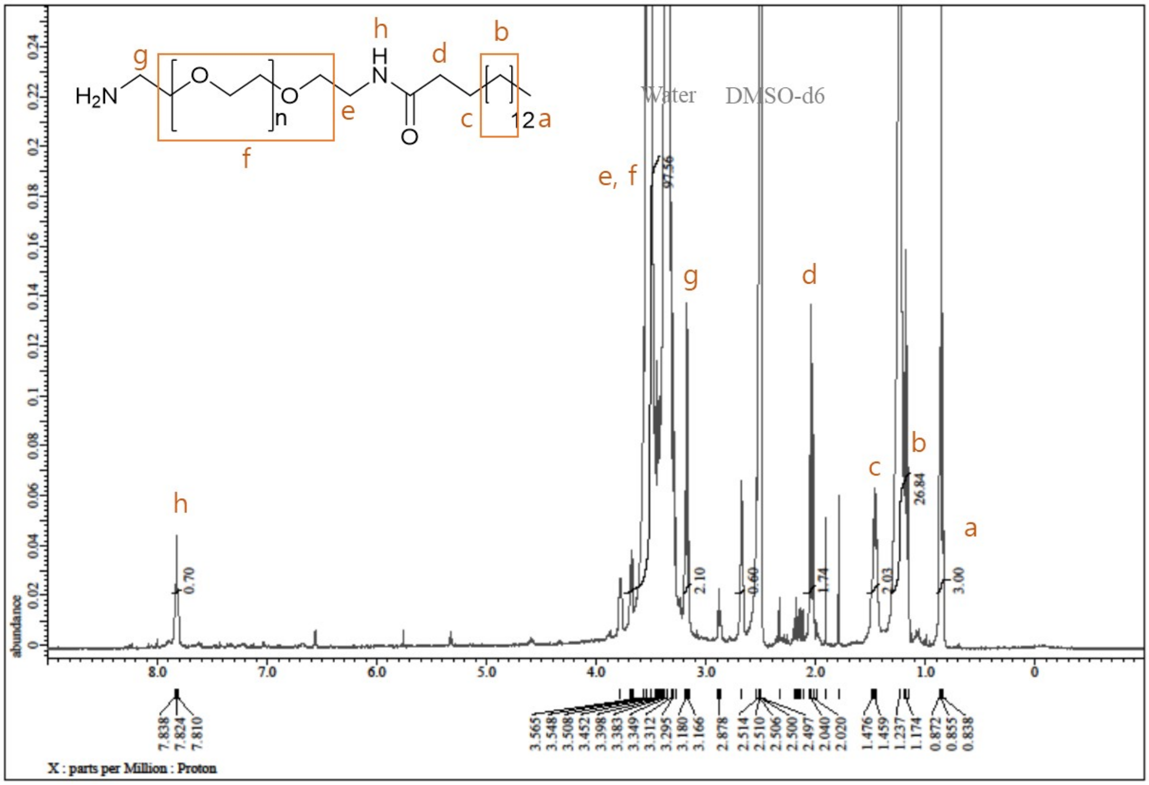

Figure S3. ^{1}H NMR spectral data of PP

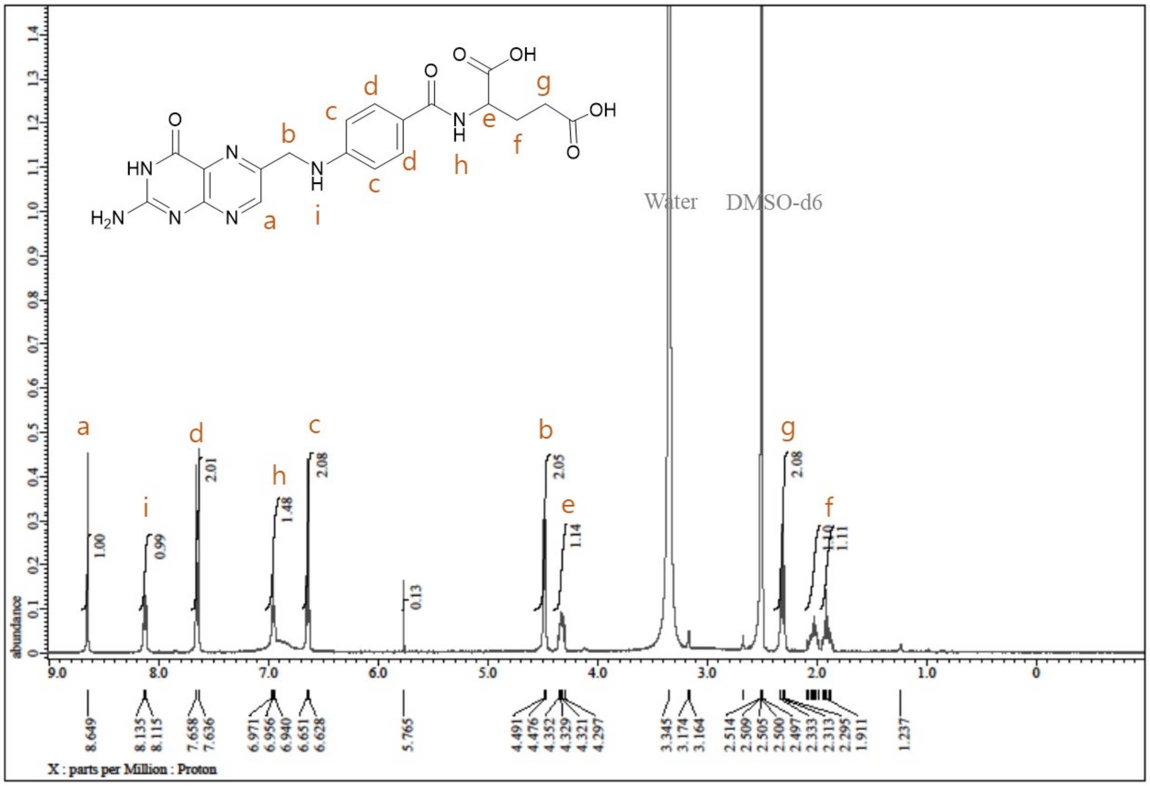

Figure S4. ^{1}H NMR spectral data of folic acid

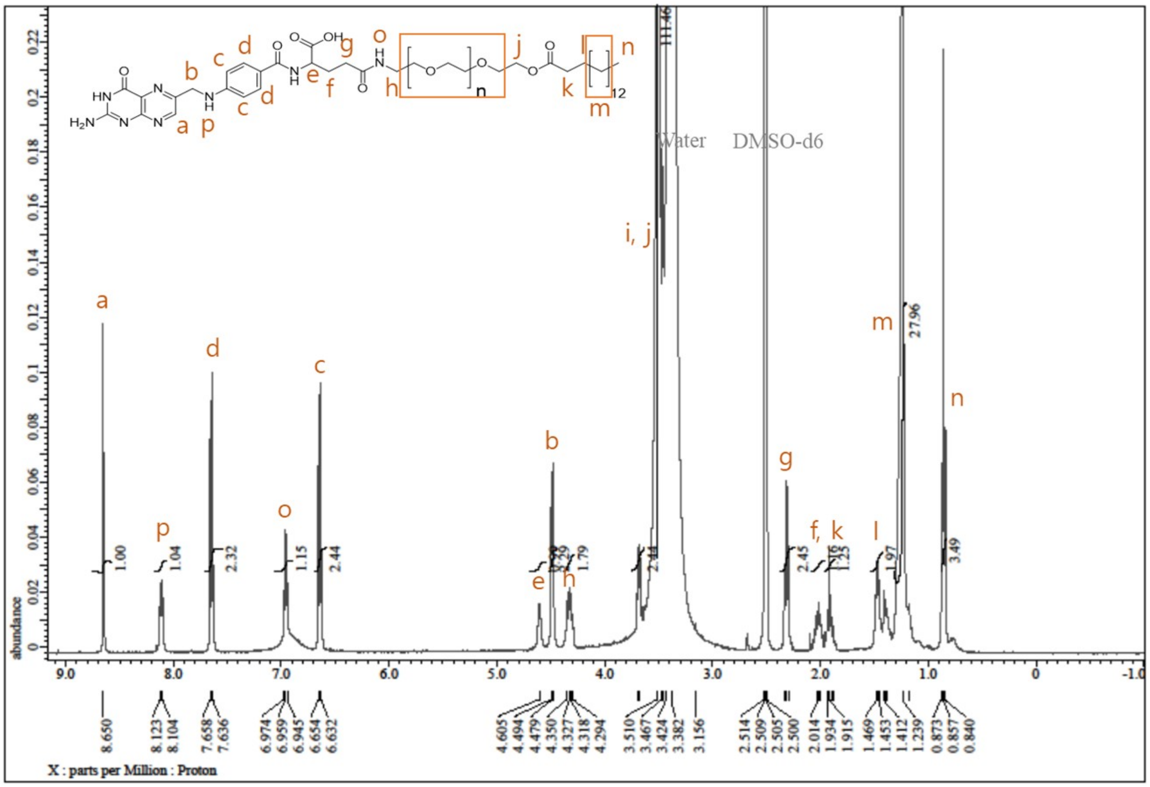

Figure S5. ^{1}H NMR spectral data of PPFA
